# Crop pangenomes

**DOI:** 10.18699/VJ21.007

**Published:** 2021-02

**Authors:** A.Yu. Pronozin, M.K. Bragina, E.A. Salina

**Affiliations:** Institute of Cytology and Genetics of Siberian Branch of the Russian Academy of Sciences, Novosibirsk, Russia; Institute of Cytology and Genetics of Siberian Branch of the Russian Academy of Sciences, Novosibirsk, Russia Kurchatov Genomic Center of the Institute of Cytology and Genetics of Siberian Branch of the Russian Academy of Sciences, Novosibirsk, Russia; Institute of Cytology and Genetics of Siberian Branch of the Russian Academy of Sciences, Novosibirsk, Russia Kurchatov Genomic Center of the Institute of Cytology and Genetics of Siberian Branch of the Russian Academy of Sciences, Novosibirsk, Russia

**Keywords:** agricultural plants, genomes, pangenomes, genes, evolution, bioinformatics analysis, computational pipelines, сельскохозяйственные растения, геномы, пангеномы, гены, эволюция, биоинформатический анализ, вычислительные конвейеры

## Abstract

Progress in genome sequencing, assembly and analysis allows for a deeper study of agricultural plants’ chromosome structures, gene identification and annotation. The published genomes of agricultural plants proved to be a valuable tool for studing gene functions and for marker-assisted and genomic selection. However, large structural genome changes, including gene copy number variations (CNVs) and gene presence/absence variations (PAVs), prevail in crops. These genomic variations play an important role in the functional set of genes and the gene composition in individuals of the same species and provide the genetic determination of the agronomically important crops properties. A high degree of genomic variation observed indicates that single reference genomes do not represent the diversity within a species, leading to the pangenome concept. The pangenome represents information about all genes in a taxon: those that are common to all taxon members and those that are variable and are partially or completely specific for particular individuals. Pangenome sequencing and analysis technologies provide a large-scale study of genomic variation and resources for an evolutionary research, functional genomics and crop breeding. This review provides an analysis of agricultural plants’ pangenome studies. Pangenome structural features, methods and programs for bioinformatic analysis of pangenomic data are described

## Abstract (ru)

Cеквенирование генома организма – важный этап в его генетических исследованиях. Расшифровка геномной последовательности открывает широкие возможности для изучения строения структуры хромосом, распределения повторенных и кодирующих последовательностей, идентификации и аннотации генов. При исследовании сельскохозяйственных растений это позволяет анализировать функции генов, разрабатывать маркеры для поиска ассоциаций с фенотипическими признаками. При решении этих задач геном вида часто представлен последовательностью одного организма (так называемым референсным геномом). В последнее время, однако, появляется много свидетельств в пользу того, что большие структурные изменения генома, включая вариации числа копий генов и вариации наличия/отсутствия генов, преобладают в сельскохозяйственных культурах, играют ключевую роль в генетическом определении агрономически важных признаков и приводят к значительным вариациям функционального набора генов и генного состава у представителей одного вида. Такие структурные вариации не могут быть представлены на основе одной лишь референсной последовательности и описываются исходя из концепции пангенома. Пангеном – это информация о полном наборе генов таксона, среди которых можно выделить набор универсальных генов, общих для всех представителей таксона, и вариабельных генов, которые являются частично или полностью специфичными для его представителей. Анализ пангеномов дает более точное понимание генетического разнообразия генофонда. Технологии секвенирования и анализа пангеномов позволяют обеспечить возможность масштабного изучения геномных вариаций, доступ к более широкому спектру геномных данных в селекционных программах и помогут ускорить селекцию культурных растений для создания сортов со стабильно высокой урожайностью и устойчивостью к стрессам. В работе представлен краткий обзор исследования пангеномов сельскохозяйственных растений, описаны их структурные особенности, методы и программы биоинформатического анализа пангеномных данных.

## Introduction

The genome sequence is the basis for a chromosome structure studying, a distribution of repetitive and coding sequences, and genes identification and annotation (Bragina et al., 2019). The different species genomes information allows a comparative phylogenetic analysis to study relationships among species, their origins, and evolutionary features (Marchant et al., 2016; Wendel et al., 2016). In agricultural plants, all these allows to assess the impact of a genetic variability on a gene function, to identify the genes responsible for the most valuable traits in crops (Schnable et al., 2009; Wing et al., 2018).

A single organism chromosome sequences serve as the basis (“reference” genome) for studying other genomes of the same species. The number of sequenced, assembled and annotated plant reference genomes increases every year (Bragina et al., 2019). The Ensembl Plants database version 48 (September 2020) contains 93 assembled and annotated plant genomes (Howe et al., 2020). Based on the reference genome sequencing and the sequencing of the same species representatives genomes (usually based on short-reading technology), genetic variability analysis, the study of the genome single-nucleotide polymorphisms (SNPs) and large structural variants (SVs) are performed. The large structural variants are the most difficult to identify using a short-read sequencing, but due to the thirdgeneration sequencing technologies (Li et al., 2018), the SVs identification is becoming more accessible and reliable. There is a growing evidence that structural variations, including copy number variations (CNVs) and presence/absence variations (PAVs), are prevalent in crops and lead to significant variations in gene content between individuals of the same species (Springer et al., 2009; Hirsch et al., 2014; Li et al., 2014; Lu et al., 2015; Zhao Q. et al., 2018).

## Genomes and pangenome

For a more efficient analysis and description of the genetic diversity, the concept of “pangenome” was proposed (Tettelin et al., 2005). The pangenome represents the information about the complete set of genes in a biological cluster (taxon), such as species, among which one can distinguish a set of universal (core) genes that are common to all organisms, and a set of unique (variable) genes that are partially shared or individually specific (Tettelin et al., 2005). Until recently pangenome studies have been focused on finding genes presence or absence in organisms to determine the universal or unique set of genes.

The concept of the “pangenome” was proposed in (Tettelin et al., 2005) for the Streptococcus agalactiae bacterial species. To date, there are several definitions of this term, wich are based on two main concepts: a function based and a structure based (Tranchant-Dubreuil et al., 2018). The structural concept considers the pangenome as complete set of taxon genomic sequences. Within this concept, taxon members genomic sequences (of the same species or genus) are compared with each other and on this basis their common unique (non-redundant) set of DNA fragments of the same length (100 bp or more, depending on the species) is determined. These sequences describe the structure of the pangenome (Snipen et al., 2009; Alcaraz et al., 2010).

The second pangenome concept is based on its functional representation. In this case, the pangenome can be described as a set of all genes for particular taxon representatives (Plissonneau et al., 2018). However, for a large number of related organisms, such a set is degenerate, because they contain a large number of genes with a high level of similarity in primary structure, and, consequently, in function. Pangenome redundancy can be eliminated by combining similar gene sequences into functional families (Sun et al., 2016). In this case, the representative genes of the same functional family in different organisms are considered as one sequence in terms of function.

The set of organisms in pangenome analysis usually limited to a single species. However, some authors use a broader interpretation of the pangenome. For example, V.V. Tetz (2003) considers the pangenome as a complete genes set of all living organisms, viruses and mobile elements.

## Pangenome structural features

Pangenome genes can be divided into two groups according to their occurence in different organisms (Golicz et al., 2016). The first group includes genes that are found in all members of the taxon. This group of genes is called the universal set or core gene set. The second group of genes includes genes that occur in a part of the taxon. This genes group is called indispensable, accessory or variable genes. Among the second genes group, the unique genes that are present only in the single individual are of particular interest. Universal and variable genes represent the functional core and the diversity of species members, respectively

From an evolutionary perspective, universal genes are mostly responsible for vital functions and they tend to be conserved within a species. In contrast, variable genes and their specific part, unique genes, contribute to the diversity of the species, enabling them to adapt to different environmental conditions. The proportion of unique genes in the studied crops pangenomes ranges from 8 to 61 % (Tao et al., 2019). However, the resulting size of the unique genome is likely to be underestimated due to the inability of current strategies and technologies to detect all functional changes in genes

**Fig. 1.**
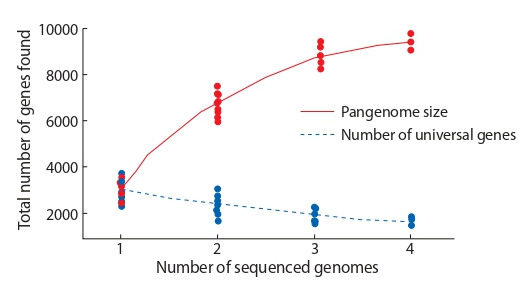
The pangenome size and the universal gene number dependence on the number of sequenced genomes

Based on the sequence of one genome it is impossible to determine, which genes are common to all species members. However, each new sequence can be assigned to a universal or variable part of the pangenome. The more taxon genomes are sequenced, the more unique genes are found. This results to a pangenome size increasing with an increase in the genomes number. However, for a universal genes set, increasing genomes number leads to the opposite result: some universal genes may be absent in other species members. As a result, the pangenome size – the set of all the different species genes – increases, while the estimated size of the universal genes set tends to decrease (Golicz et al., 2016; Wang et al., 2018). This relation is shown schematically in Fig. 1. Each point on the graph corresponds to an estimate of the genes number in the pangenome for a set of k genomes (taken randomly from the full sample of N genomes under study). With k increasing, the estimate of the total pangenome genes number increases (red line), and the unique genes number decreases (blue dashed line). Examples of dependencies for real pangenomes can be found at https://pangp.zhaopage.com. Thereby, the organisms sample sizes significantly affects the pangenome size estimation and the universal gene proportion in it.

In addition to the sequenced genomes number, the pangenome unique gene size and proportion is also influenced by many factors. The choice of a sample for analysis is one of them: (1) wild and cultivated species together will give a larger pangenome with a higher percentage of unique genes than only cultivated plants (Montenegro et al., 2017; Zhao Q. et al., 2018); (2) the ploidy level, mode of reproduction, bottlenecks during domestications, etc. A plant species with higher levels of ploidy and outbreeding and reduced diversity because of domestication tend to have a higher percentage of unique genes (Tao et al., 2019).

It can be assumed that the addition of an unlimited number of new genomes to the pangenome could lead to its unlimited growth. However, the gene diversity studies in crop species have shown the number of unique genes decrease as the number of sequenced samples increases. This suggests that, given a certain number of taxon representatives, the inclusion of additional genomes in the pangenome will no lead to a further increase in its genes number. Such pangenomes are called “closed”. The “closed” pangenome was found in tomato (Gao et al., 2019), corn (Hirsch et al., 2014), rice (Wang et al., 2018), soybeans (Li et al., 2014), sunflower (Hübner et al., 2019), Brachypodium distachyon (Gordon et al., 2017), Brassica napus (Hurgobin et al., 2018) and Brassica oleracea (Golicz et al., 2016). 

However, there are also “open” pangenomes, in which the total genes number grows with each new sample added. Open pangenomes are specific for microorganisms, for example for the wheat leaves septoria fungal pathogen Zymoseptoria tritici (Plissonneau et al., 2018). The bacterium Paenibacillus polymyxa pangenome also belongs to the open type (Zhou et al., 2020).

**Fig. 2.**
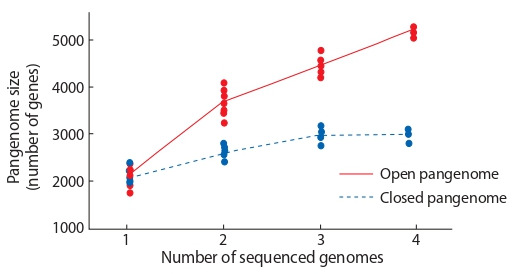
The dependence of the genes number in the pangenome (Y-axis) from the number of sequenced taxon representatives (X-axis) for two pangenome types: open and closed. For open genomes, number of genes raise monotonically, for closed – reaches a plateau.

If organisms from the population are randomly selected, the pangenom type can be estimated by plotting the number of found genes in each new genomic sequence (Fig. 2). The pangenome genes number reaching a plateau after analysis of certain genomic sequences number characterizes “closed” pangenomes (see Fig. 2, blue dashed line). The “open” pangenomes are characterized by a constant increase in size when new genomes are added (see Fig. 2, red line).

The comparison of the pangenome size and the universal and variable pangenome parts for some plant species is shown in (Supplimentary 1)^1^. The data obtained demonstrates the number of samples for pangenome analysis varies from three (B. rapa) to three thousand (Oryza sativa). The genes number in pangenomes varies from 35 thousand in diploid rice to 128 thousand in hexaploid bread wheat. The proportion of universal genes ranged from 41 % in Мedicago truncatula to 84 % in B. rapa.

^1^ Supplementary materials 1–3 are available in the online version of the paper: http://www.bionet.nsc.ru/vogis/download/pict-2021-25/appx2.pdf


## Pangenome functional features

Researches show that universal genes are responsible for fundamental cellular processes, while variable genes are associated primarily with functions that can give an advantage in different environmental conditions. Thus, Brachypodium distachyon pangenome analysis demonstrated universal gene set annotations are enriched with terms such as “glycolysis”, “steroid”, “glycosylation”, “co-enzyme” (Gordon et al., 2017). Variable genes sets annotations were most of all enriched with terms “protective function”, “development”. In the same work, it was shown the nonsynonymous/synonymous substitution rate ratio in variable genes are higher than in universal genes. In addition, the universal genes orthologs in rice and sorghum were found to be more conservative than orthologs of the variable genes set. Universal genes expression level is generally higher than variable genes (Gordon et al., 2017). Similar results were obtained in the soybeans (Li et al., 2014; Liu et al., 2020), cabbage (Golicz et al., 2016), and wheat (Montenegro et al., 2017) pangenomes analysis.

The analysis of several agricultural plant pangenomes showed (Tao et al., 2019) that (1) the variable genes sequences are more mutable than universal genes; (2) the nonsynonymous substitution rate ratio is higher in variable genes; (3) variable genes are characterized by a wide function diversity; (4) the variable and universal genes functional characteristics are different, the variable genes are more related to the response to environmental factors, receptor activity and signal transduction, the universal genes are more related to basic cellular functions. Thus, the universal genes represent the conservative core of the pangenome (and species, respectively), while the variable genes represent its mutable part (both in terms of function and in terms of primary structure and expression patterns). 

## Pangenomes and pantranscriptomes

The transcriptome analysis is another gene set analysing method in several members of a taxon. The transcript nucleotide sequences (mainly mRNA), their expression levels estimation and the isoforms presence can be obtained by high-throughput sequencing (RNA-seq), which is significantly cheaper than the genome sequencing. Transcriptomic data allows estimating genes presence in the genome only if they are expressed in a plant tissue or organ. Thus, a set of transcripts cannot represent the full genome gene composition, but it is possible to obtain an approximate estimation (especially if a transcripts set from different tissues at different stages of development is analyzed). In this case, the transcriptome assembly requires significantly less computational resources, and the current methods allow obtaining it with high quality

A study of the 503 inbred maize lines pantranscriptome revealed genetic diversity in protein-coding genes: more than 1.5 million single-nucleotide variations were found, and mutations associated with plant development traits (timing of several growth phases) were identified (Hirsch et al., 2014). 

M. Jin et al. (2016) also analysed the 368 inbred maize lines pantranscriptome. The analysis identified more than two thousand sequences that were not represented in the maize reference genome, including genes responsible for the biotic stress response. Variations that are associated with the gene expression level (eQTL) were analysed. The analysis’ results were projected to metabolic networks, which allowed to specify their functioning mechanisms.

Y. Ma et al. (2019) analysed 288 barley transcriptome sequencing experiments. Among the collected transcripts, about 30 % showed no similarity to the reference genome. The results of the pantranscriptome analysis revealed that pathogen resistance genes are more numerous in wild-grown barley, and such genes were subjected to greater selection pressure during domestication compared to genes in other species.

## Pangenome construction methods

The pangenome bioinformatic analysis can be divided into the following main steps:

- The pangenome sequence assembling.
- The conserved and variable genomic sequences regions identification.
- Genes identification/prediction and functional annotation.
- Polymorphisms identification.
- Storage, rapid access and visualization of the pangenomic data.

The following pangenome assembly strategies exist: assembly-alignment; metagenome approach; mapping-assembly (Golicz et al., 2016; Hurgobin, Edwards, 2017; TranchantDubreuil et al., 2018).

**Assembly-then-map.** This strategy consists of each taxon separately de novo assembly, followed by sequences alignment with each other as well as with the reference genome to decrease redundancy and identify a set of common and variable sequence regions. Several software packages have been developed for the genome assembly: Velvet (Zerbino et al., 2008), SOAPdenovo (Xie et al., 2014), ALLPATHS (Butler et al., 2008) and MaSuRCA (Zimin et al., 2013). This approach is time-consuming and computationally intensive. The de novo assembly strategy has been used for the pangenome analysis of cultivated soybean (Li et al., 2010), wild soybean (Li et al., 2014), rice (Wang et al., 2018), B. oleracea (Golicz et al., 2016) and Medicago truncatula (Zhou et al., 2020).

**Metagenomic-like approach.** This strategy consists to all sequenced fragments from different taxon representatives combining into one pool and the de novo assembling pangenome sequences from these fragments. Each assembled contig is then assigned to a particular genome by the sample original reads alignment to the metagenomic assembly and then contig coverage is evaluated. This method allows lowcoverage sequencing results to be handled. The metagenomic approach has been used to analyse the genome of rice (Yao et al., 2015) and tomato (Gao et al., 2019).

**Map-then-assembly.** This strategy uses one complete genome assembly (reference sequence) as the basis for the genome assembly of the other taxon members (guide assembly). The reads from a single species are mapped to the reference genome, and not mapped reads are discarded and assembled separately. The reference genome sequence is complemented with new sequences, and the samples are compared with the reference genome. This method reduces the time required to construct a pangenome. If a genomic segment is found in more than one sample, the segment will be integrated from the first sample while the de novo method creates two complete genomes. This strategy has been used in the sunflower pangenome analysis (Hübner et al., 2019).

It should also be noted, that in a number of studies, the researchers did not use the genomic sequences assembly, but aligned short reads to a reference genome. This approach allows assessing the SNP and phenotypic plants characteristics relations. Methods based on the short reads alignment are also described, which allows the identification of structural rearrangements, duplications and gene losses (Zhao et al., 2013). The alignment method was used in the maize pantranscriptome analysis (Hirsch et al., 2014), in the assessment of CNV’s changes in the potato pangenome analysis (Żmieńko et al., 2014).

## Pangenome analysis and annotation methods

Based on a comparison of sequences, genome annotation allows identifying gene sequences in taxon representatives’ genomes, to determine orthologous genes and universal and variable genes families. Several software packages are designed for pangenomes automatic annotation. They perform the main steps of the pangenome sequence analysis and annotation. The capabilities of a number of these programs are briefly described below.

**PGAP** (Zhao Y. et al., 2012) performs large-scale gene search, functional annotation, orthologous gene clusters ontology term enrichment, species evolution analysis, pangenome structural analysis, and the universal and variable pangenome parts identification. In the updated version of this program, PGAP-X (Zhao Y. et al., 2018), methods for presentation and visualization of pangenome analysis results are further developed.

**PpsPCP** (Tahir ul Qamar et al., 2019) was developed for a pangenome PAV identification. The analysis is based on a full-genome taxon and a reference genome sequences comparison in several rounds with sequential correction of both gene set and gene alignment sites in the reference genome. As a result, a pangenome gene set is created by combining the individual genome sequences with the reference genome and their annotation.

**BPGA** (Chaudhari et al., 2019) provides a wide range of pangenome analysis opportunities: gene clustering based on sequence similarity, orthologs presence/absence analysis, the pangenome and its universal part sizes plotting, phylogenetic tree reconstruction, metabolic pathway and functional annotation analysis, GC composition deviation assessment, various statistical pangenome characteristics calculation, and several other features.

**panX** (Ding et al., 2018) aims to identify orthologous genes clusters. The sequence comparison clustering, verification and refinement of cluster composition based on evolutionary distance analysis and phylogenetic reconstruction, and assesses the association between the gene composition of individual taxon members and their phenotypes are used.

**Pan4Draft** (Veras et al., 2018) is designed to improve pangenome annotation by adding sequence information on unfinished genomes. An annotation and assembly to the chromosome level in these genomes is incomplete, but their sequences contain genomic DNA fragments and provide valuable information about the species genome diversity. Information about plant pangenome analysis methods and software for processing and analysis of plant pangenome are provided in Supplementary 2 and 3.

## Pangenomic data use perspectives

Currently, the research field of the crop pangenomes sequencing and analysis is developed rapidly and provides more and more information about genetic variations and new genes. 

One of the fundamental problems in the crop pangenomes study is to evaluate the genetic diversity of their cultivated representatives as well as wild relatives. This analysis allows us to establish the origin and evolution of cultivated plants, to estimate the breeding process impact on the genetic structure of varieties. Thus, the pangenome analysis helps to answer a number of important questions about patterns of the genome evolution at species level, about mechanisms of the genes de novo origination, the gene functions diversity and their associations with phenotypic traits of plants.

One of the important directions of the crop pangenome research is the wild relatives’ genome sequencing and analysis. It is supposed that wild relatives of cultivated plants may contain a pool of genes related to adaptation of organisms to environmental conditions, response to biotic stresses; i. e. those genes that may have been lost by cultivated plants as a result of artificial selection (bottleneck effect) (Goncharov, Kondratenko, 2008; Goncharov, 2013; Purugganan, 2019). The discovered genes can be further used to create new genotypes that are more resistant to pathogens, pests and abiotic stress. Thus, the study of agricultural plant pangenomes has not only a fundamental aspect, but is also important in terms of practical breeding

## Conclusion

A better understanding of genetic diversity, combined with advanced sequencing technologies and high-throughput phenotyping can facilitate trait analysis to identify useful genetic mutations. In addition, it allows to access a wider range of genetic resources helps to select the best strategies in breeding programmes and ultimately accelerates crop breeding to develop varieties with consistently high yields under stressful conditions.

Pangenomic studies offer a wider understanding of the crop gene pools genetic diversity than genome resequencing studies and thus can be extremely useful for the crop improvement. Nevertheless, the knowledge obtained through pangenomic researches requires integration with QTL/GWAS and genome resequencing studies to identify important genes and alleles to be used in an effective breeding strategy.

## Conflict of interest

The authors declare no conflict of interest.
